# Breast density does not impact the ability of Videssa^®^ Breast to detect breast cancer in women under age 50

**DOI:** 10.1371/journal.pone.0186198

**Published:** 2017-10-25

**Authors:** David E. Reese, Meredith C. Henderson, Michael Silver, Rao Mulpuri, Elias Letsios, Quynh Tran, Judith K. Wolf

**Affiliations:** Provista Diagnostics, New York, NY, United States of America; Fondazione IRCCS Istituto Nazionale dei Tumori, ITALY

## Abstract

Breast density is associated with reduced imaging resolution in the detection of breast cancer. A biochemical approach that is not affected by density would provide an important tool to healthcare professionals who are managing women with dense breasts and suspicious imaging findings. Videssa^®^ Breast is a combinatorial proteomic biomarker assay (CPBA), comprised of Serum Protein Biomarkers (SPB) and Tumor Associated Autoantibodies (TAAb) integrated with patient-specific clinical data to produce a diagnostic score that reliably detects breast cancer (BC) as an adjunctive tool to imaging. The performance of Videssa^®^ Breast was evaluated in the dense (*a* and *b*) and non-dense (*c* and *d*) groups in a population of n = 545 women under age 50. The sensitivity and specificity in the dense breast group were calculated to be 88.9% and 81.2%, respectively, and 92.3% and 86.6%, respectively, for the non-dense group. No significant differences were observed in the sensitivity (p = 1.0) or specificity (p = 0.18) between these groups. The NPV was 99.3% and 99.1% in non-dense and dense groups, respectively. Unlike imaging, Videssa^®^ Breast does not appear to be impacted by breast density; it can effectively detect breast cancer in women with dense and non-dense breasts alike. Thus, Videssa^®^ Breast provides a powerful tool for healthcare providers when women with dense breasts present with challenging imaging findings. In addition, Videssa^®^ Breast provides assurance to women with dense breasts that they do not have breast cancer, reducing further anxiety in this higher risk patient population.

## Background

Imaging is currently the gold standard for breast cancer detection. When imaging results are questionable (scored as Breast Imaging-Reporting and Data System (BI-RADS) 3 or 4), NCCN Guidelines recommend that BI-RADS 3 are followed with re-imaging at 6 months, while BI-RADS 4 are recommended for biopsy [1]. Confounding factors such as breast density may limit the effectiveness of imaging [2–4]. Breast density is a radiologic phenomenon that is not discernable by palpation; it is a radiologist’s assessment. Imaging rays permeate dense breast tissue in a more opaque manner, thereby reducing image resolution [5]. Density is subdivided into four categories: (*a*) almost entirely fatty (10% of women); (*b*) scattered areas of fibroglandular density (40%); (*c*) heterogenously dense (40%); and (*d*) extremely dense (10%). African American women tend to have higher breast density than women of European descent [6]. Women with very dense breasts are at a four- to five-fold increased risk of developing breast cancer compared to women with low breast density [2,7,8]. In addition, women with extremely dense breasts are 10 times more likely to have an abnormality missed on mammogram [1].

Thirty-two U.S. states have laws mandating that women be notified of the implications of breast density to encourage discussions between patients and health-care providers regarding the need for supplemental screening [9–11]. Of the 32 states currently mandating notifications, 21 mention the association with increased cancer risk and 17 mention supplemental screening, of which only four mention specific screening modalities. This lack of consistency leads to confusion for healthcare providers in the management of women with dense breasts and their follow-up. In addition, the notification of increased breast cancer risk can increase anxiety in women with a dense breast assessment.

Recently, a study evaluated the differential screening performance of digital mammography combined with tomosynthesis compared to digital mammography alone as a function of breast density [12]. When tomosynthesis was combined with digital mammography for screening, the cancer detection rate increased (by ~20%) and recall rates decreased (by ~13%) for women with dense and non-dense breasts. These improvements were most pronounced in women with heterogeneously dense breasts but were not significant for women with extremely dense breasts. Therefore, imaging limitations persist in the dense breast patient population despite advancements made by tomosynthesis. A separate study evaluated the inter-observer precision of scoring breast density in a group of ten mammography technologists and seven radiologists [13]. This study identified that mammography technologists over-grade breast density, which can result in redundant ultrasound studies, unnecessary breast biopsies, increased costs, and patient anxiety. A biochemical approach that is independent of breast architecture and density would provide an important tool to healthcare professionals who are managing women with dense breasts.

Recently, clinical performance data was published on Videssa^®^ Breast, a CPBA comprised of SPB and TAAb integrated with clinical characteristic data to produce one diagnostic score that reliably detects breast cancer [14]. Certain blood-based biomarkers are associated with higher mammographic density [15]; it is unknown whether the biomarkers included in Videssa^®^ Breast might be impacted as well. The objective of this study was to evaluate the performance of Videssa^®^ Breast in women with dense and non-dense breasts and determine whether this test could provide an additional tool to healthcare providers managing women who present with dense breasts and challenging imaging findings.

## Methods

### Study design and participants

Provista-001 (Clinicaltrials.gov, NCT01839045) and Provista-002 (Clinicaltrials.gov, NCT02078570), which were sponsored by Provista Diagnostics, enrolled women categorized as either BI-RADS 3 or 4 at the time of enrollment. All imaging modalities such as mammography, 3D tomography, ultrasound and MRI were permitted in the trial for the assessment of BI-RADS 3 or 4. Participants were enrolled at 13 clinical sites across the US, and the study was IRB-approved at all of these sites (S1 Table). All participants provided written informed consent. Participants who were not diagnosed with BC were followed for six or twelve months for clinical outcomes, which included additional imaging or pathology results. Samples were collected between 04/2013–06/2014 for Provista-001 and between 04/2014–02/2015 for Provista-002.

Breast density status was retrospectively collected for all participants according to the American College of Radiology reporting system [16]:

1. The breasts are almost entirely fatty
2. There are scattered areas of fibroglandular density
3. The breasts are heterogeneously dense, which may obscure small masses
4. The breasts are extremely dense, which lowers the sensitivity of mammography

In this study, “dense” breasts were defined as being assigned Category *c* or *d* and “non-dense” breasts as Category *a* or *b*.

### Measurement of SPB and TAAb in serum

Serum was tested for SPB and TAAb as previously described [14]. Briefly, SPB plates were purchased from Meso Scale Discovery (MSD; Rockville, MD) and processed as per manufacturer instructions. Recombinant TAAb proteins were purchased from Origene (Rockville, MD) and Abnova (Walnut, CA) and coated on standard-bind MSD 384-well plates. Samples were analyzed for the relative presence or absence of TAAb by indirect ELISA. All plates were read using a Meso Sector S600 plate reader and Discovery Workbench 4.0 software.

### Videssa^®^ Breast assessment

Serum was tested with Videssa^®^ Breast as previously described [14]. Briefly, SPB and TAAb data are combined with patient age into a logistic regression algorithm, the final output being a high protein signature (HPS) or low protein signature (LPS). The protein signature outcome corresponds to the likelihood of BC at the time of sample collection. BC samples scored as HPS were categorized as true positive (TP) and BC samples scored as LPS were categorized as false negative (FN). Benign samples scored as HPS were categorized as false positive (FP) and benign samples scored as LPS were categorized as true negative (TN).

### Statistical analyses

Clinical performance metrics were calculated using the following:

Sensitivity = (True Positives) ÷ (True Positives + False Negatives)
Specificity = (True Negatives) ÷ (True Negatives + False Positives)
PPV = (True Positives) ÷ (True Positives + False Positives)
NPV = (True Negatives) ÷ (True Negatives + False Negatives)

All analyses were conducted using SAS (version 9.3; SAS, Cary, NC) and the 95% confidence intervals were calculated using VassarStats, which utilizes the efficient-score method (corrected for continuity) [17,18].

## Results

### Study population

**Fig 1:**
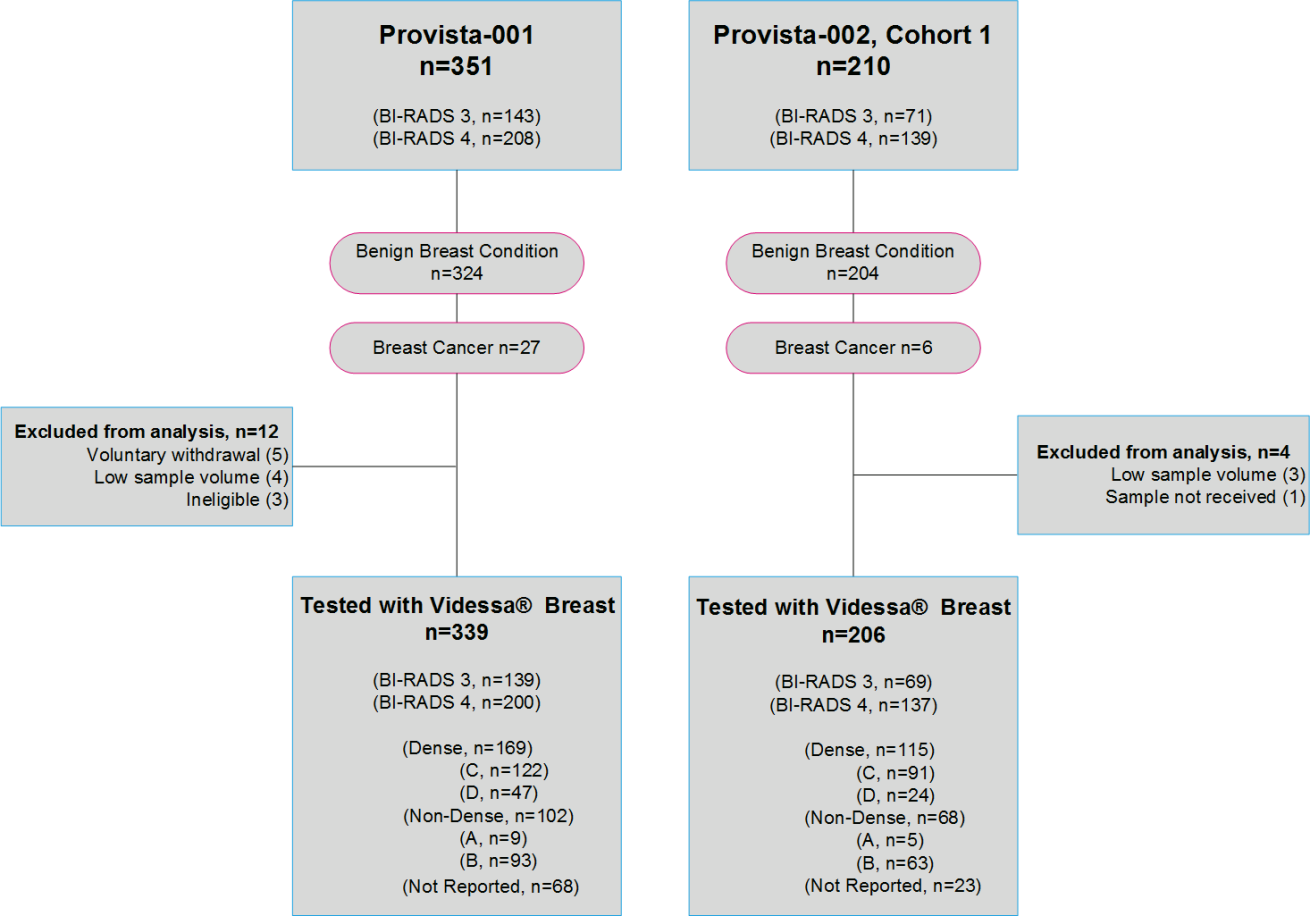
Flowchart detailing numbers for enrolled subjects, subjects included in the current study, and numbers in each arm. Exclusion criteria included screen failures (subjects not within the trial’s stated inclusion criteria), withdrawal of consent during study, and/or low sample volume (< 2 mL).

An overview of the Provista-001 and Provista-002 clinical trials is provided in Fig 1. In Provista-001, there were 339 eligible participants tested with Videssa^®^ Breast. Of these, 313 participants were diagnosed with a benign breast condition (includes participants diagnosed by biopsy and participants not diagnosed with BC during the follow-up period). Twenty-six participants were diagnosed as having breast cancer (includes invasive and DCIS). In Provista-002, a total of 206 participants were tested with Videssa^®^ Breast. Of these, 200 participants were diagnosed with a benign breast condition and six participants were diagnosed as having breast cancer. Participant characteristics are provided in Table 1.

**Table 1:** Participant Demographics.

		Clinical Study			
		Provista-001		Provista-002Cohort One	
**n =**		339		206	
		
**Age—Median**		43		44	
**Range (Min-Max)**		(26–49)		(26–49)	
		
**Race**					
White		266	*79%*	162	*79%*
Black/African American		18	*5%*	23	*11%*
Asian		15	*4%*	7	*3%*
American Indian / Alaska Native/Hawaiian / Pacific Islander		5	*2%*	5	*3%*
Other*		35	*10%*	9	*4%*
**Ethnicity**					
Hispanic or Latino		37	*11%*	22	*11%*
Not Hispanic or Latino**		302	*89%*	184	*89%*
		
**BI-RADS**					
	**3**	139	*41%*	69	*33%*
	**4**	200	*59%*	137	*67%*
**Breast Density**					
**Non-dense**		102		68	
**a**		(9)		(5)	
**b**		(93)		(63)	
**Dense**		169		115	
**c**		(122)		(91)	
**d**		(47)		(24)	
**Status Not Available**		68		23	
		
**Benign Breast Condition**		313		200	
Pathology Confirmed Benign		(166)		(122)	
Presumed Benign***		(145)		(76)	
Lobular carcinoma *in situ*‡ (LCIS)		(2)		(2)	
		
**Breast Cancer**		26		6	
Invasive carcinoma (IBC)		(18)		(2)	
Ductal carcinoma *in situ* (DCIS)		(8)		(4)	

* Multicultural or not reported

** Includes participants that did not report ethnicity

*** Presumed all non-cancer participants to be benign

‡ LCIS participants were categorized as non-cancer (benign)

In the complete Provista-001 and Provista-002 retrospective set scored with Videssa^®^ Breast, there were 284 women with dense breasts and 170 women with non-dense breasts as defined by American College of Radiology [19].

### Evaluation of individual Videssa^®^ Breast biomarkers in women with dense and non-dense breasts

The individual biomarkers included in Videssa^®^ Breast were evaluated in the dense and non-dense groups to determine whether there were any individual biomarkers that were differentially expressed between these groups. These data are summarized in Table 2. A total of four biomarkers demonstrated statistically significant (p < 0.05) differences in expression (IL-6, IL-8, ErbB2, and HGF). Videssa^®^ Breast biomarker data are combined in an algorithm along clinical patient information, thus these differences do not necessarily suggest a bias in Videssa^®^ Breast performance in terms of breast density.

**Table 2:** Comparison of biomarker expression in dense versus non-dense populations.

Biomarker Type	Abbreviation	Full Name	p-value*
SPB	IL-6	Interleukin-6	0.0102
SPB	IL-8	Interleukin-8	0.0495
SPB	TNF-α	Tumor necrosis factor	0.7279
SPB	CEA	Carcinoembryonic antigen	0.2813
SPB	ErbB2	Receptor tyrosine-protein kinase erbB-2	< .0001
SPB	OPN	Osteopontin	0.1095
SPB	HGF	Hepatocyte growth factor receptor	< .0001
SPB	VEGF-C	Vascular endothelial growth factor C	0.8099
TAAb	FRS3	Fibroblast growth factor receptor substrate 3	0.6511
TAAb	GPR157	Probable G-protein coupled receptor 157	0.699
TAAb	HOXD1	Homeobox protein Hox-D1	0.4235
TAAb	p53	Cellular tumor antigen p53	0.6563
TAAb	PDCD6IP	Programmed cell death 6-interacting protein	0.9282
TAAb	SELL	L-selectin	0.4800
TAAb	SERPINH1	Serine Proteinase Inhibitor H1	0.9779
TAAb	SF3A1	Splicing factor 3A subunit 1	0.7108
TAAb	TFCP2	Alpha-globin transcription factor CP2	0.7447
TAAb	TRIM32	E3 ubiquitin-protein ligase TRIM32	0.6046

*Fisher exact test used to determine p-value.

### Evaluation of Videssa^®^ Breast in women with dense and non-dense breasts

To understand the performance of Videssa^®^ Breast in women with dense breasts, the clinical sensitivity, specificity, NPV and PPV were evaluated in the dense and non-dense groups from the comprehensive Provista-001 and Provista-002 set (n = 545). Of these, breast density information was available for 454; 62.6% (n = 284) were categorized as having dense breasts (*c* and *d*) and 37.4% (n = 170) were categorized as having non-dense breasts (*a* and *b*). These performance data are provided in Table 3. The sensitivity of Videssa^®^ Breast in the non-dense and dense groups was 92.3% and 88.9%, respectively, and the specificity in the non-dense and dense groups was 86.6% and 81.2%, respectively. No significant differences were observed in the sensitivity (p = 1.0) or specificity (p = 0.18) of Videssa^®^ Breast in detecting breast cancer in participants with non-dense breasts and dense breasts. The NPV in both groups exceeded 99%; PPV was slightly higher (though not statistically significant) in the non-dense group (36.4% and 24.2% for non-dense and dense breasts, respectively).

**Table 3:** Videssa^*®*^ Breast clinical performance metrics in women with dense and non-dense breasts. The n = for each subpopulation (a, b, c, and d) are shown in parentheses below the total n = for the main groups (TP, TN, FP, and FN). 95% confidence intervals (CI) are shown in parentheses below each clinical performance measure. Prior and posterior probabilities are given for non-dense and dense breast subjects if test outcome is positive (HPS- high protein signature) or negative (LPS- low protein signature).

Breast Density Category† (n = 545)	TP	TN	FP	FN	Sens.	Spec.	NPV	PPV	Test Probability
**Non-Dense (n = 170)**	**12**	**136**	**21**	**1**	**92.3%**(62–99%)	**86.6%**(80–91%)	**99.3%**(95–100%)	**36.4%**(21–55%)	**Prior (8%)**
***a***	(1)	(13)	(0)	(0)					**(Post- HPS) 36%**
***b***	(11)	(123)	(21)	(1)					**(Post- LPS) 1%**

**Dense (n = 284)**	**16**	**216**	**50**	**2**	**88.9%**(64–98%)	**81.2%**(76–86%)	**99.1%**(96–100%)	**24.2%**(15–37%)	**Prior (6%)**
***c***	(11)	(161)	(39)	(2)					**(Post- HPS) 24%**
***d***	(5)	(55)	(11)	(0)					**(Post- LPS) 1%**

Not Recorded (n = 91*)	0	78	12	1	**p = 1.00** **	**p = 0.18** **			

* Breast density information was not available for 91 participants

† Dense breast categories are as defined by American College of Radiology [Sickles et al., BI-RADS Atlas]

**Two-tailed Fisher Exact Test comparing TP&FN (sensitivity) and TN&FP (specificity) in dense and non-dense subgroups.

## Discussion

Dense breast tissue has been shown to significantly impact the ability of imaging modalities to detect breast cancer lesions, resulting in increased false-negatives and false-positives [2,20]. Often, clinically, dense breast tissue results in additional imaging above and beyond standard mammography [12]. Furthermore, confusion is likely when women are informed of a dense breast assessment but are not provided with clinical direction. Women who are informed that they have dense breast tissue may believe this to be a clinically significant finding, akin to a benign condition. Recent advances in imaging have attempted to improve imaging in dense breast patients with limited success [12]. A protein-based diagnostic assay that is unaffected by density would be a powerful non-invasive tool to compliment current imaging technologies.

While individual protein biomarker expression levels have been shown to be altered by breast density, it was unclear whether a biomarker panel composed of many individual biomarkers would be affected. An interesting observation from this study is the lack of statistical difference in dense verses non-dense for TAAb, but not SPB. While some SPB did show statistically significant differences, Videssa^®^ Breast demonstrated high sensitivity in women with non-dense and dense breasts (92.3% and 88.9%, respectively). This test also exhibited high specificity in both groups, 86.6% and 81.2%, respectively. There were no significant differences observed in the sensitivity or specificity of the Videssa^®^ Breast between patients with non-dense and dense breasts. NPV exceeded 99% in both groups, but this figure may be skewed due to low disease prevalence. Prior test probability was 8% for non-dense and 6% for dense breast subjects (Table 3), based on BC prevalence in the study population. Posterior probability for a negative (LPS) test outcome was 1% for both groups, meaning a negative test will almost always correspond to the subject being free of breast cancer. Therefore, unlike imaging, Videssa^®^ Breast is not impacted by breast density; it can reliably detect breast cancer in women under age 50 with dense and non-dense breasts. While additional studies are needed to determine validity in other age/clinical groups, these results strongly argue for the use of an anatomical assessment (imaging) in conjunction with an assay (biologic/protein measurement), such as Videssa^®^ Breast, in women with dense breasts.

The population studied were all subjects with either benign, DCIS, or cancerous findings. A large number of benign subjects (221/513, or 43%) did not undergo biopsy, therefore clinical sensitivity and specificity may have been slightly underestimated. Breast cancer prevalence is generally low in women under age 50 (5.9% in this study), thus an enriched population (BI-RADS 3 and 4) was necessary. Because Videssa^®^ Breast is indicated only for BI-RADS 3 or 4, this study was also a robust test of assay performance in the intended use population. Because of Videssa^®^ Breast’s high negative predictive value, there is likely value in ruling out cancer in women with dense breasts, who may experience higher recall rates. The incorporation of Videssa^®^ Breast into the clinical decision-making process has the potential to decrease medical costs associated with additional imaging and biopsy while simultaneously calming the anxiety that women with dense breasts may experience with imaging alone.

## Conclusions

This study demonstrates that Videssa^®^ Breast performance is comparable in women with dense and non-dense breasts. Videssa^®^ Breast demonstrates high sensitivity and specificity in detecting breast cancer, irrespective of density status. Thus, Videssa^®^ Breast provides an additional tool for health-care providers when women with dense breasts present with challenging imaging findings. In addition, a negative Videssa^®^ Breast test provides assurance to women with dense breasts that they likely do not have breast cancer, thereby reducing anxiety in this higher risk patient population.

## Declarations

### Ethics approval and consent to participate

This study was approved by the IRB governing all clinical sites that enrolled patients in this trial, the names of which are included in S1 Table. All participants were provided with informed consent and agreed to study participation prior to sample collection. Full list of all site-specific IRB: Avera Cancer Institute IRB, Rhode Island Hospital IRB, Scripps Cancer Center IRB, Henry Ford Health System IRB, Chesapeake IRB, Lahey Hospital Medical Center IRB, Mercy Health IRB, Dignity Health St. Joseph's IRB, Western IRB, Mayo IRB.

## Supporting information

S1 Table Clinical study sites. Some sites participated in both the Provista-001 and Provista-002 studies, these are noted in the first column.
(DOCX)

S2 Table Full line data for comparison of Videssa^®^ Breast performance in women wi*th dense and non-dense breasts*. (XLSX)
