# Supplementary material for: Breast density does not impact the ability of Videssa® Breast to detect breast cancer in women under age 50
Source: PLoS One. 2017 Oct 25;12(10):e0186198. doi: 10.1371/journal.pone.0186198 (PMC5656317; doi:10.1371/journal.pone.0186198)
Supplement: S1 Table — Some sites participated in both the Provista-001 and Provista-002 studies, these are noted in the first column. (DOCX) [file pone.0186198.s001.docx]

| **Clinical Trial** | **Institution** | **City** | **State** | **IRB** |
| --- | --- | --- | --- | --- |
| Provista-001/ Provista-002 | Avera Cancer Institute | Sioux Falls | South Dakota | Avera Cancer Institute |
| Provista-001/ Provista-002 | Rhode Island Hospital | Providence | Rhode Island | Rhode Island Hospital |
| Provista-001/ Provista-002 | Scripps Cancer Clinic | San Diego | California | Scripps Cancer Center |
| Provista-001/ Provista-002 | Henry Ford Hospital | Detroit | Michigan | Henry Ford Health System |
| Provista-001/ Provista-002 | Sutter Institute for Medical Research | Sacramento | California | Chesapeake IRB |
| Provista-001 | Banner Research | Phoenix | Arizona | Chesapeake IRB |
| Provista-001 | Lahey Clinic | Peabody | Massachusetts | Lahey Hospital Medical Center |
| Provista-001 | Sansum Clinic | Santa Barbara | California | Chesapeake IRB |
| Provista-002 | Mercy Oncology Center | Oklahoma City | Oklahoma | Mercy Health |
| Provista-002 | St. Joseph’s Hospital | Phoenix | Arizona | Dignity Health St. Joseph |
| Provista-002 | Sinai Grace Detroit Medical Center | Detroit | Michigan | Western IRB |
| Provista-002 | Mayo Clinic Scottsdale | Scottsdale | Arizona | Mayo IRB |
| Provista-002 | Mayo Clinic Rochester | Rochester | Minnesota | Mayo IRB |
